# Wearable Music Tuning Ring for the Concert Pitch Using PVDF/BCZT Piezoelectric Composites

**DOI:** 10.3390/polym18141739

**Published:** 2026-07-16

**Authors:** Mengchen Zhou, Tian Zhang

**Affiliations:** 1 Department of Musicology, Wuhan Conservatory of Music, Wuhan 430060, China; 2 Electronic Information School, Wuhan University, Wuhan 430072, China

**Keywords:** wearable music electronics, standard pitch, tuning, PVDF, signal processing

## Abstract

Instrument tuning is crucial for the performance quality of orchestral concerts. In most cases, the standard pitch is usually given by the oboist, and then the rest of the orchestra adjusts their instruments to match it. However, in the individual training scene, music instrument players often rely on a piano or a pitch pipe for the standard pitch. The piano is not portable, and the pitch pipe could be inaccurate. Therefore, a portable, accurate and user-friendly tuning device is urgently required. In this work, we sought to design a wearable music ring using high-performance PVDF/BCZT composites to stimulate the standard pitch when needed anywhere and anytime. In the music ring, there are two core buttons that stimulate and modulate the output voltage and create the standard pitch. To optimize the performance, we constructed a composite structure, with BCZT particles embedded in highly oriented polyvinylidene fluoride (PVDF) fibers. The composite presents an aligned fiber structure, which endows it with high piezoelectricity. It presents sensitivity as high as 0.12 V N^−1^ and remains stable after 10,000 cycles at 1 Hz. Furthermore, the volume of the standard pitch could be modulated by switching the buttons on the wearable music ring, which meets real application needs. This research presents a protocol showing a typical application of wearable technology, which not only shapes the development of flexible electronics but also redefines the tuning experience for musicians.

## 1. Introduction

The mutual conversion between mechanical energy and charge serves as the core characteristic of piezoelectric materials [1,2,3,4,5]. Self-powered pressure sensors based on the piezoelectric effect exhibit capability for real-time pressure and vibration detection, thus showing potential applications in many fields, such as wearable electronics, health monitoring, human robots, etc. [6,7,8,9,10]. Many potential applications have been considered in past studies, yet most cases remain in the imagination. New applications should consider and address real existing problems [11,12,13]. In the music field, there is an important operation before the performance of an orchestra, which is the standard pitch proofread. Currently, the oboe is utilized to produce the standard pitch, and it must last more than 10 s to guarantee all the players can adjust their musical instruments. This situation is acceptable during orchestra performances. However, when players practice individually, especially for string players, obtaining the standard pitch is a typical issue [14,15].

In this work, we design and fabricate a wearable music ring that could produce the standard pitch (also called concert pitch (A = 440 Hz)), thereby addressing a long-standing issue in this field. Based on application requirements, the wearable music ring should be flexible and comfortable for long-time usage [16,17]. It is well known that piezoelectric composites have the advantages of both the high piezoelectricity of ceramics and the excellent flexibility of the polymer matrix [18,19,20]. PVDF is a typical piezoelectric polymer, which exhibits five crystalline phases, among which the β phase is the one with the highest piezoelectricity. The transformation from the α to the β phase is pursued for applications in electromechanical conversion [21,22]. A series of strategies have been proposed, among which electrospinning is a universal approach for enhancing the β-phase content and, in turn, achieving high piezoelectricity, due to the intrinsic high stretching and electric field during fiber preparation [23,24,25]. In addition, the incorporation of inorganic fillers and conductive additives into the PVDF matrix is another method to enhance the piezoelectricity. As a typical lead-free piezoelectric ceramic, BCZT exhibits a piezoelectric coefficient up to 620 pC/N and a Curie temperature of around 120 °C [26,27]. Thus, we chose BCZT as the ceramic phase in this work. Some previous studies have focused on PVDF/BCZT composites to investigate the correlation between preparation, microstructure and electric properties [28,29,30].

Based on the above statement, we designed a wearable music ring based on PVDF/BCZT composites fabricated via the electrospinning approach. BCZT particles were fabricated via the traditional solid-state method, and different amounts were added to the PVDF matrix. By adjusting the rolling speed, piezoelectric fibers with aligned structures were obtained. The higher the rolling speed, the higher the alignment that could be achieved. The mechanical stability was evaluated using a long-time cycling test to confirm its capability for wearable applications. The prepared wearable music ring can be comfortably attached to human fingers, and its size can be easily adjusted to meet requirements. With fingers pressing on the wearable music ring, the standard pitch was then produced. This work was carried out under real-world scenarios, shaping the future development of wearable electronics.

## 2. Experimental Section

### 2.1. Preparation of PVDF/BCZT Fibers

Ba_0.85_Ca_0.15_Zr_0.10_Ti_0.90_O_3_ ceramics were prepared via a conventional solid-state reaction method. Analytical-grade oxide powders (BaCO_3_, CaCO_3_, ZrO_2_, TiO_2_) were provided by Sinopharm Chemical Reagent Co. Ltd. (Shanghai, China), which serve as the starting materials. They were first weighted according to the nominal compositions and then planetary ball milled in a polyethylene jar with ZrO_2_ balls for 12 h using deionized water as the liquid medium. Following that, the prepared slurry was dried thoroughly and calcined at 1250 °C for 3 h. Then, the pre-sintered powders were ball milled again and sintered at 1450 °C for 4 h to obtain high-crystalline powders. The sintered powders were sieved with 200-mesh sieve for following use, without another treatment.

N,N-Dimethylformamide (C_3_H_7_NO, DMF) and acetone (CH_3_COCH_3_) were supplied by Sinopharm Chemical Reagent Co. Ltd. (Shanghai, China). PVDF powder was purchased from PolyK (State College, PA, USA). It was dissolved in mixed solution of DMF and acetone and then stirred at room temperature until a clear solution was obtained. BCZT particles were added in the solution and stirred for another 12 h to guarantee the uniform dispersion of ceramics in polymer matrix. The composite solution was stored in a plastic syringe with a 21G stainless-steel needle for the following electrospinning process. We set the feed rate of the syringe as 0.15 mm/min, applied positive voltage, and negative voltage was set as 12 and −2 kV, respectively. The rotation speed of the drum collector covered with aluminum foil was set as 2800 rpm to obtain highly aligned PVDF/BCZT fibers. To act as a comparison, we utilized two other rotation speeds (300 and 1400 rpm) to investigate their influence on the alignment of fibers. Finally, the obtained PVDF/BCZT fibers were fully dried at 60 °C for 12 h to remove the residual solvent. The obtained fibers were named PVDF/BCZT-*y* (*y* is the mass fraction of BCZT, which is set as 10 wt%). The fibers were encapsulated in polyimide for environmental stability.

### 2.2. Characterizations

X-ray diffraction (XRD): Crystal structures of BCZT particles and PVDF/BCZT composites were analyzed using Cu Kα radiation X-ray diffraction (λ = 1.5406 Å, operated at 40 kV and 20 mA, Rigaku Smartlab, Tokyo, Japan). Scanning Electron Microscopy (SEM) and Energy-Dispersive X-ray Spectroscopy (EDS): Cross-sectional morphologies of composites and BCZT were characterized using field-emission SEM (JSM-7610FPlus, Rigaku, Tokyo, Japan) at 5 kV. Samples were sputter-coated with around 5 nm Au prior to imaging. We tested the sample using a thickness gauge (CHY-CA, Jinan Saicheng Electronic Technology Co. LTD., Jinan, China). Thickness measurements were performed at 5 random positions (center and four corners) of each sample to avoid errors caused by uneven thickness. FTIR analysis: Fourier-transform infrared (FTIR) spectra of the composite films were acquired using a Nicolet 6700 spectrometer (FT-IR, Nicolet6700, Bruker Spectrometer, Saarbrücken, Germany) in transmission mode with a spectral resolution of 4 cm^−1^ over a range of 700–1300 cm^−1^. Poling process was conducted to improve the piezoelectricity of fibers. The poling field was set as 10 kV/mm, and time duration was 15 min.

## 3. Results and Discussion

### Preparation and Characterization of BCZT and PVDF/BCZT Composites

**Figure 1:**
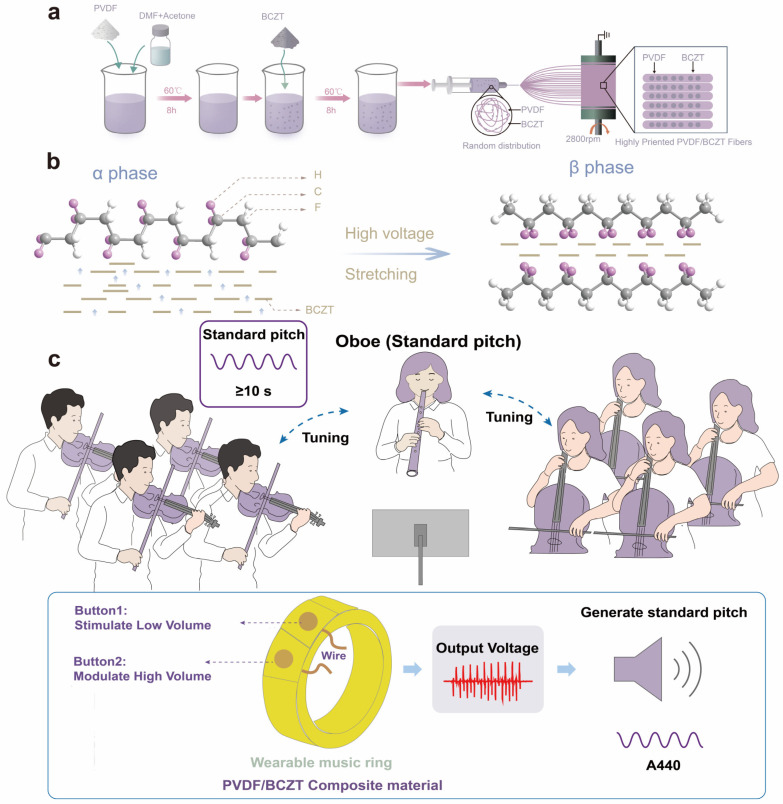
The preparation flowchart of the PVDF/BCZT composites and demonstration of their applications. (**a**) Schematic diagram of the preparation of composites. (**b**) Interaction between the PVDF and the BCZT particles. (**c**) Schematic diagram of the standard pitch production and tuning process.

A preparation flowchart of BCZT particles and PVDF/BCZT composites is shown in Figure 1. BCZT is a kind of promising lead-free piezoelectric ceramic, with high piezoelectricity and Curie temperature. In addition, its composition did not have volatile elements, which guarantees performance consistency. This work adopts solid-state reaction method to synthesize BCZT particles, with two-step sintering employed to boost crystallization. PVDF powders were dissolved in DMF/acetone mixed solvent, and BCZT particles of different mass fractions were added for the following electrospinning process. To guarantee the highly aligned fiber structure, we set the rotating speed as 2800 rpm. The higher the rotating speed, the higher the stretching force, which improves the alignment of electrospun fibers. BCZT particles were wrapped in PVDF fibers; thus, both high piezoelectricity and benign flexibility can be obtained in PVDF/BCZT composites. As is well known, the α is the most stable phase in the PVDF matrix without piezoelectricity. Thus, various processing methods (hot pressing, electrospinning, stretching, etc.) have been proposed to induce the effective transformation from the α to the β phase [17,18]. The transition mechanism is given in Figure 1b, with high voltage and stretching force included. Figure 1c illustrates the working principle and application scenarios of a wearable musical tuning ring system based on the PVDF/BCZT composite. The system aims to generate a stable standard pitch through wearable technology, providing real-time tuning support for players and enhancing pitch consistency. As shown in the diagram, normally, prior to a concert performance, the oboist first produces a concert pitch A with a duration of no less than 10 s to serve as the tuning benchmark for the orchestra. Specifically, in collaborative multiplayer scenarios, different musicians (such as violin, viola and cello) subsequently adjust their open strings in response, thereby achieving overall pitch unification. In an individual training scene, players rely on a tuning device. For individual practice, however, players depend on tuning devices, which often lack real-time interactivity, portability, and ergonomic comfort during extended use, motivating the development of a wearable alternative. The lower section of the figure presents the overall device architecture and signal output mechanism of the wearable musical ring. The ring integrates functional modules based on PVDF/BCZT composite piezoelectric material and adopts a dual-button control system: Button 1 corresponds to low volume, while Button 2 corresponds to high volume. When triggered, the piezoelectric composite converts mechanical stimuli into stable electrical signals, which subsequently drive the acoustic module to produce the standard pitch, thereby realizing the portable and real-time instrument tuning assistance function. The electrical signals were first transmitted to the signal processing module and then to the sound generation module.

**Figure 2:**
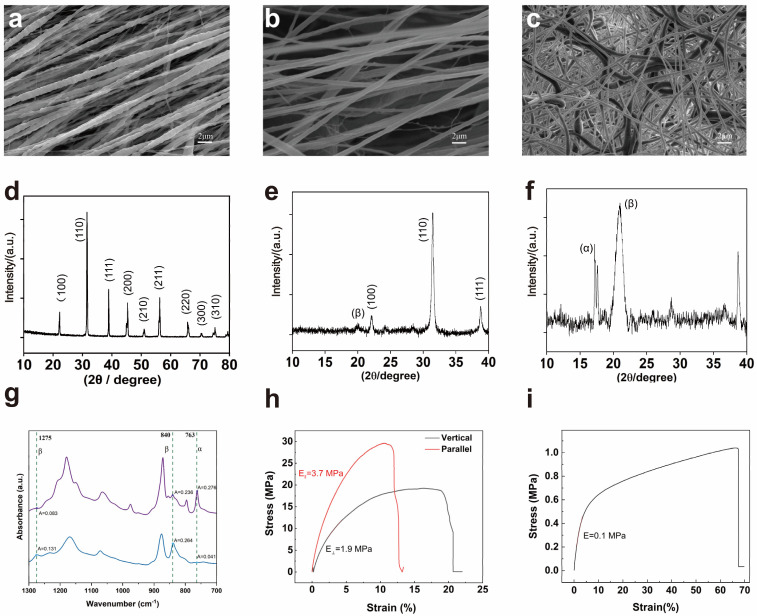
Characterization of prepared PVDF/BCZT composites. (**a**–**c**) SEM images of the PVDF/BCZT fibers prepared by electrospinning process with different rotating speeds (2800, 1400 and 300 rpm); (**d**–**f**) XRD patterns of the BCZT particles, the PVDF/BCZT composites and the pure PVDF matrix; (**g**) FTIR spectrum of the PVDF/BCZT before and after electrospinning process (the upper line and lower line correspond to before and after electrospinning process, respectively); (**h**,**i**) stress–strain curves of the composite fibers vertical and parallel with alignment direction.

SEM images with different alignments are shown in Figure 2a–c, originating from the distinct rotating speed of the drum collector. The alignment degree of fibers rises linearly with increasing rotating speed. In this study, the fibers with the highest alignment degree were selected for the following investigation. The X-ray diffraction pattern of the BCZT, the PVDF and the PVDF/BCZT composites is shown in Figure 2d–f. It is observed that no obvious secondary impurity phase could be traced within the sensitivity of the instrument. BCZT mainly presents a tetragonal phase, which is well demonstrated by the splitting peak at 45° [20].

In the electrospinning process, the high electric field and mechanical stretching help to promote the transformation from the α phase to the β phase [21,23]. There is a significant increase in the β-phase content after the electrospinning process. The β-phase content reached up to 81% based on the calculation. As is well known, the characteristic absorption bands of the α phase were indexed at 763 cm^−1^ and 976 cm^−1^, while those of the β phase were characterized by 840 cm^−1^ and 1279 cm^−1^. According to the Beer–Lambert equation, the relative fraction of the β phase is given by Equation (1)

(1) $$F\left(\beta \right)=\frac{A_{\beta }}{{(K}_{\beta }/K_{\alpha })A_{\alpha }+A_{\beta }}$$

where F(β) is the proportion of the β phase in the crystal region; A_α_ and A_β_ are the absorbances at 764 and 840 cm^−1^; and K_α_ and K_β_ are the absorption coefficients at the wavenumbers, which are 6.1 × 10^4^ and 7.7 × 10^4^ cm^2^ mol^−1^, respectively. During the electrospinning process, the strong electrostatic tension makes the PVDF fibers arrange along the polar direction, which promotes the formation of the β phase. These results demonstrate that the electric field and mechanical alignment during fiber fabrication trigger a conformational transition of the PVDF molecular chains from the nonpolar TGTG′ conformation to the polar all-trans (TTTT) conformation, which further promotes the generation of the β phase.

Figure 2h presents the stress–strain curves of the prepared composite fibers along the parallel and vertical directions, exhibiting distinct anisotropic mechanical responses. The composite fibers were pressed at 10 MPa and room temperature to guarantee the mechanical performance. When stretched along the parallel direction, the composite fibers deliver higher mechanical strength, with a maximum tensile stress of approximately ~30 MPa and a Young’s modulus of about ~3.7 MPa. In contrast, the vertical direction exhibits maximum stress of only ~18 MPa and a Young’s modulus of ~1.9 MPa. These results indicate that the fibers possess superior load-bearing capacity and rigidity along their alignment direction. This phenomenon is primarily attributed to the formation of a pronounced oriented arrangement structure during fiber fabrication. When an external force is applied along the parallel direction, stress can be effectively transferred across the continuously aligned fiber network, enhancing load distribution capability and structural stability. By contrast, interfacial slippage between fibers and localized stress concentration readily emerges in the perpendicular direction, leading to overall deteriorated mechanical performance. Furthermore, the parallel direction displays higher peak stress accompanied by lower fracture strain, indicating that the material exhibits high strength and stiffness yet reduced ductility. As a counterpart, the vertical direction demonstrates a lower modulus and more gradual deformation behavior. Figure 2i further illustrates the stress–strain relationship of the composite fibers, with the maximum fracture strain reaching approximately 65% and a corresponding elastic modulus of about 0.1 MPa. In the low-strain regime, the curve demonstrates a gradual linear increase, suggesting excellent elastic deformation capability. As the strain increases, stress progressively rises and eventually stabilizes, reflecting that the internal network structure undergoes fiber rearrangement.

**Figure 3:**
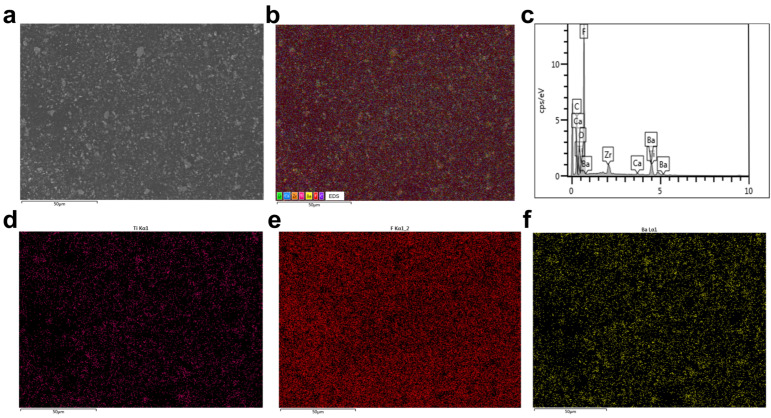
EDS mapping results. (**a**) Surface morphology of PVDF/BCZT composites; (**b**) element distribution of all the elements (Ba, Ca, Zr, Ti, O, C and F); (**c**) weight percent of all elements; (**d**–**f**) Ti, F and Ba elements to show the even distribution of BCZT in PVDF matrix.

As mentioned above, we change the rotating speed (300, 1400 and 2800 rpm) to achieve aligned PVDF fibers with BCZT particles embedded. As can be concluded, 2800 rpm is the best parameter for fully aligning the fibers. The diameter of fibers is around 0.2–1 μm, and higher speed induces smaller diameter due to the high stretching force. We investigate the mechanical properties of PVDF/BCZT composites with different ceramic contents. Overall, variations in BCZT loading exert a limited influence on the Young’s modulus. By contrast, the Young’s modulus along the fiber alignment is higher than that in the perpendicular direction. This phenomenon is reasonable as the adjacent fibers exhibit a weak bond, which is much lower than the intrinsic tensile strength of individual fibers. The element distribution was established via EDS mapping, with the results shown in Figure 3. BCZT particles were evenly distributed in the PVDF matrix. Ba and Ti were selected as the characteristic elements of BCZT particles, and F was chosen as the characteristic element in the PVDF matrix.

**Figure 4:**
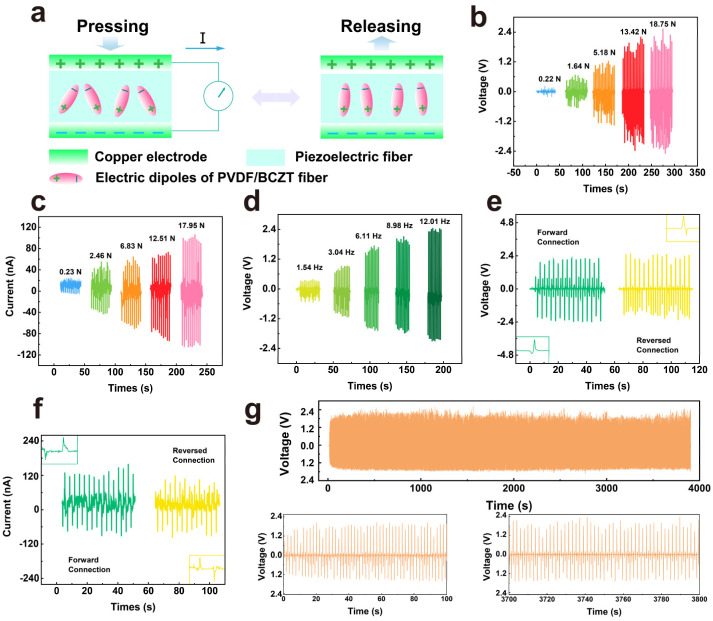
Electrical performance of PVDF/BCZT composites. (**a**) Working mechanism under press-and-release process; (**b**,**c**) output voltage and current at various pressures; (**d**) output voltage and current at various frequencies; (**e**,**f**) forward and reverse connection; (**g**) long-term stability of the prepared wearable music ring, with two enlarged views to show the details.

To evaluate the piezoelectric properties of composites, a series of devices with distinct BCZT contents were prepared (Figure 4). Figure 4a illustrates the intrinsic mechanism of flexible sensors, where the voltage/current could be generated under external mechanical stress. The conversion between stress and electricity originated from the rotation of dipoles, thus inducing charge accumulation in up and bottom electrodes. We investigated the output voltage and current under different pressures; increased pressure induces higher output. Note that electrospinning fibers are utilized for device preparation without a poling procedure. Forward and reverse connection serves as a feasible method to confirm that the signal is really from the piezoelectric device rather than environmental noise or triboelectric interference [15,28]. The output voltage and current were both inversed with roughly equal magnitude, indicating that these electrical signals were generated by an intrinsic piezoelectric response (Figure 4e,f). The electromechanical performance was characterized under different frequencies. As can be seen, the output voltage varies significantly with excitation frequency, which is in line with previous studies. For real applications, the long-term durability is indispensable. The negligible variation after 10,000 cycles confirms the excellent stability of the device for long-term use.

**Figure 5:**
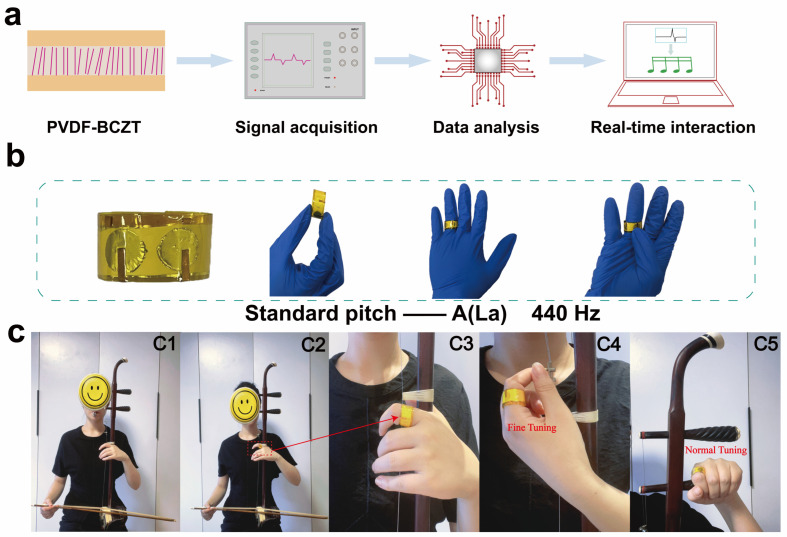
The design concept and working principle of wearable music ring. (**a**) Working flowchart of wearable music ring, from the composites to signal acquisition, data analysis and real-time interaction; (**b**) images of prepared wearable music ring and attachment on finger; (**c**) demonstration of wearable music ring for standard pitch by a musician to play the traditional Chinese string instrument Erhu.

The design configuration of the wearable music ring is given in Figure 5a, which involves signal acquisition, data analysis, real-time interaction, etc. The PVDF/BCZT composites were encapsulated in PET sheets to protect the devices and guarantee the consistency of electrical performance. We attached the wearable music ring to the finger for use, as given in Figure 5b. The standard pitch was produced when the wearable ring was pressed by the thumb. Once the wearable music ring is pressed, as discussed above, the PVDF/BCZT composites would stimulate the voltage and current output. Through data analysis, the real interaction was activated to produce standard pitch. For the designed device, there are two buttons on it, with one for low volume and one for high volume. Wearing the designed wearable music ring, the player could use their thumb to switch buttons so as to adjust the volume of the standard pitch, which is more convenient than traditional counterparts. To demonstrate the real application of the wearable music ring, we use the Chinese traditional music instrument Erhu as an example. The Erhu, a traditional Chinese two-string instrument played with a bow, similarly requires pitch adjustment prior to a performance or practice, following the same tuning principle as Western string instruments. For the Erhu instrument, there are two kinds of tuning approach, namely fine tuning and normal tuning. Fine tuning is performed by adjusting the Erhu fine tuner. In detail, the fine tuner is usually installed on the string. It could be possible to fulfill fine adjustments by rotating its gear carefully, thus achieving precise pitch calibration. Normal tuning is conducted using tuning pegs. By rotating the tuning pegs, the string tension is adjusted accordingly, thus achieving large-scale pitch calibration. The kind of tuning approach adopted depends on the deviation from the standard pitch. In some cases, both a fine tuner and tuning pegs are utilized for better adjustment. Figure 5(c1) demonstrates the tuning process with a pitch pipe, which serves as a widely adopted tool for routine Erhu training and practice. In this study, we propose to use a wearable music ring to serve as an alternative, which is more comfortable and user-friendly. When the Erhu player presses the button on the ring, the voltage is then produced and, in turn, transmitted to the signal processing module and finally to the sound generation module (producing the sound of concert pitch A). When the note is produced, the player starts the tuning process.

The demonstration with the Erhu confirms the feasibility of the designed wearable music ring for the tuning process in individual training. Compared with the piano, the wearable music ring is more accessible and portable. Compared with the pitch pipe, the ring outperforms it due to the intrinsic advantages of pitch accuracy and reliability, ease of operation and aesthetic appeal [31]. Firstly, the wearable music ring ensures reliable pitch accuracy. A pitch pipe relies on airflow to produce sound, and its pitch is significantly influenced by factors such as the player’s breath intensity. Different players may produce slightly different pitches, and pitch shifts may appear even with the same player, depending on how hard they blow. The proposed wearable music ring utilizes a standard acoustic module to produce standard pitch directly, which is not affected by how the user plays it. It outputs an accurate, stable standard pitch (A 440 Hz) each time it is triggered, basically getting rid of the pitch uncertainty that comes from human playing. Secondly, the tuning process is optimized. The pitch pipe demands the player to use breathing techniques to produce sound, which imposes a higher skill requirement on the user; specifically, it continuously engages their breath control, thereby reducing their focus on pitch tuning. The proposed device is triggered by pressing with a player’s fingertips, eliminating any breathing techniques. It lowers the barrier to use and lets players focus all their attention on pitch. Furthermore, because the device is worn on the finger, hands stay free while playing, allowing the player to rotate the tuning pegs at the same time. This enables simultaneous listening and tuning without interruption, thus streamlining the whole tuning process. Thirdly, the device integrates practical functionality with aesthetic appeal. Traditional pitch pipes are prone to being lost or forgotten in mobile performance settings. By contrast, the proposed device worn on the finger stays with the user at all times, effectively avoiding the risk of misplacement. More importantly, the ring configuration endows the tuning ring with ornamental value—its form, size, and color can be customized to reflect the user’s personal aesthetic, transforming it from a purely functional tool into a work of aesthetic expression. In summary, the proposed design outperforms traditional pitch pipes in terms of pitch accuracy, user experience, and aesthetic value, offering a more efficient, elegant, and reliable solution for instrument tuning.

## 4. Conclusions

In this study, we designed and prepared a high-performance PVDF/BCZT composite via the electrospinning process. Then, the composites were constructed as a wearable music ring for producing standard pitch, which is widely demanded in individual music training scenes. We investigated the electrical performance of composites under different parameters and correlated them with the microstructure. Standard pitch with different volumes was successfully demonstrated by pressing the buttons on the device, which utilized the sensing capability of piezoelectric PVDF/BCZT composites. A demonstration of the wearable music ring was conducted with a traditional Chinese string instrument, Erhu. This work shapes the future development of wearable electronics in music training scenes.

## Data Availability

The raw data supporting the conclusions of this article will be made available by the authors on request.
